# Effects of Tempering on Microstructure and Properties of Additive Manufacturing Cu-Bearing AISI 431 Steel

**DOI:** 10.3390/ma17184628

**Published:** 2024-09-21

**Authors:** Li Zhao, Baichun Li, Chaolin Tan, Hongmei Zhu

**Affiliations:** 1 Key Laboratory of Hunan Province of Equipment Safety Service Technology under Extreme Environment, Hengyang 421001, China; liliya0218@126.com (L.Z.); libaichun106@126.com (B.L.); 2 Singapore Institute of Manufacturing Technology (SIMTech), Agency for Science, Technology and Research (A*STAR), 5 Cleantech Loop, Singapore 636732, Singapore

**Keywords:** laser-directed energy deposition, martensitic stainless steel, tempering time, microstructure, mechanical properties

## Abstract

AISI 431 martensitic stainless steels (MSS) with 2.5 wt% Cu were fabricated via laser-directed energy deposition additive manufacturing followed by single-step tempering treatment. The influences of different tempering times at 600 °C on microstructure and mechanical properties of the as-deposited 431-2.5Cu MSS have been explored and analyzed. The as-deposited MSS specimen primarily consisted of lath martensite, austenite and *M*_23_C_6_ carbide. After the single-step tempering treatment at 600 °C, Cu-enriched (ԑ-Cu) nano-precipitates and reverse austenite can be formed and promoted by extending the tempering treatment. The microhardness, strength and elongation can be improved with increasing the tempering time up to 1.0 h, and subsequently reduced with the tempering time prolonging to 2.0 h. Compared to 431 MSS that requires a multiple-step heat treatment for excellent performance, the 431-2.5Cu MSS specimen presented superior tensile properties after single-step tempering at 600 °C for 1.0 h in the present work. The ultimate tensile strength (UTS), yield strength (YS) and elongation (EL) of one-hour tempered MSS were 1611 MPa, 1334 MPa and 16.3%, respectively. This study provides a quantitative theoretical reference and experimental basis for realizing short-process fabrication of the Cu-bearing MSS with high strength and ductility.

## 1. Introduction

AISI 431 martensitic stainless steel (MSS) has been broadly applied in aerospace and marine engineering, owing to its inherent merits including high strength, high ductility and moderate corrosion resistance [1,2]. However, the further industrial applications of conventionally cast 431 MSS have been restricted due to the following two main aspects: (i) the poor weldability; and (ii) the requirement for a two-step heat treatment of 950–1050 °C quenching and subsequent 275–350 °C tempering [1].

To realize the manufacturing and remanufacturing of large-scale complex MSS components with high mechanical properties, laser-directed energy deposition (LDED) technology has gradually been utilized [3,4,5,6,7]. To date, the majority of studies have focused on the effects of laser parameters [2,8,9,10] and alloying elements [11,12] on laser fabricating 431 MSS. For example, Wang et al. [11] revealed that the microhardness of the 431 MSS decreased with the increase in Mo contents (0–6 wt%) due to the reduction in the martensite/ferrite ratio, but the wear resistance was obviously enhanced owing to the refined microstructure and the generated M_2_C precipitates. Recently, Liu et al. [1] investigated the effects of the post-heat treatment process on microstructure and mechanical properties of the LDEDed 431 MSS. It was found that the optimized UTS (1283 MPa) and EL (14.5%) were achieved after a three-step heat treatment (including a 680 °C × 2.0 h tempering, 1050 °C × 45 min solid-solution treatment and 315 °C × 3.0 h tempering) [1].

It has been well demonstrated that mechanical properties of cast MSS can be greatly improved via the Cu addition and subsequent suitable heat treatment, due to the precipitation strengthening effect induced by the Cu-enriched phase [13,14,15,16,17]. For instance, Ye et al. [17] investigated the influences of Cu content (0, 1.5 and 3 wt%) on tensile properties of the cast 15%Cr MSS after the solid-solution treatment at 1050 °C for 30 min and tempering treatment at 600 °C for 2.0 h, and obtained an increment of 62% in the product of strength and elongation (PSE) for 3 wt% Cu MSS in contrast to the Cu-free MSS. Furthermore, Huang et al. [18] reported that the addition of 4 wt% Cu improved the Rockwell hardness of 420 MSS, which can be attributed to the coexistence of carbides and ԑ-Cu particles after solid-solution treatment at 1040 °C for 30 min.

Nevertheless, there is a limited amount of literature exploring the influences of post-heat treatment on the microstructure and mechanical properties of laser-fabricated 431 MSS [1]. Our previous work has verified that the optimal mechanical properties of LDEDed 431 MSS could be obtained at a Cu content level of 2.5 wt% [12]. Hence, this work focused on the post-LDED heat treatments of the MSS, using single-step tempering at 600 °C under different time conditions (0.5 h, 1.0 h and 2.0 h). The influences of tempering time on the microstructural evolution and mechanical properties of the LDEDed MSS specimen were carefully investigated. The present work highlights the pathway for achieving high performance in MSS by combining Cu alloying and streamlined post-heat treatment.

## 2. Materials and Methods

### 2.1. Materials and LDED Process

Spherical 431 MSS and Cu powders, provided by China Changsha TIJO Metal Materials Co., Ltd., were mechanically mixed and then dried with an average size of 75 µm; these were used as feedstocks for the LDED fabrication. A36 mild steel was used as the substrate. The nominal compositions of 431-2.5wt%Cu MSS powder were 0.11 C, 16.5 Cr, 1 Si, 1 Mn, 2.7 Ni, 2.5 Cu, and balance Fe (in wt%).

**Figure 1:**
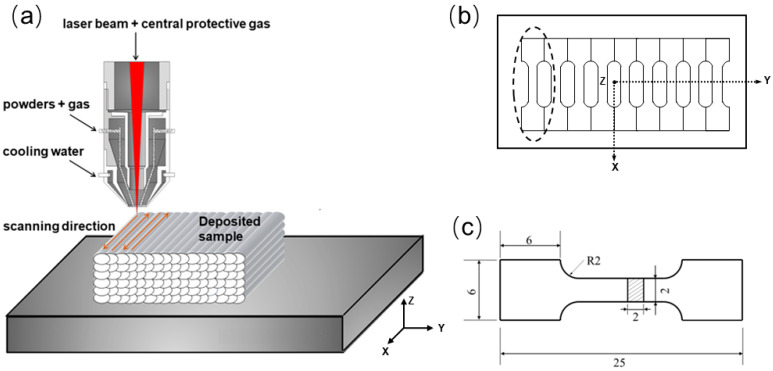
LDED process and tensile specimen. (**a**) Laser processing; (**b**) sampling and (**c**) geometry of tensile specimen.

Ten-layer specimens with dimensions of 90 mm × 40 mm × 4 mm were fabricated with an FL-1500 1.5 kW fiber laser containing a coaxial powder feed and water-cooling system (Figure 1a). Based on our previous work, the laser power density of 430 W/mm^2^, scanning speed of 480 mm/min, overlap rate of 50%, and powder delivery rate of 6.5 g/min were utilized. During the deposition, nitrogen gas with a purity of 99.999% and a flow rate of 10 L/min was used as both the shielding and carrier gas.

### 2.2. Heat Treatment

**Figure 2:**
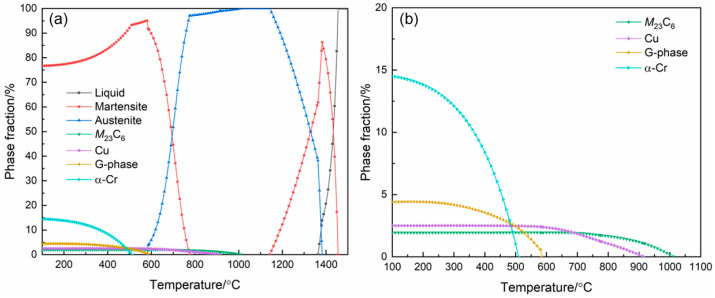
Full equilibrium phase diagram in the temperature range of 100 to 1500 °C (**a**) and partial equilibrium phase fractions in the temperature range of 100 to 1100 °C (**b**).

To ascertain the tempering temperature, the equilibrium phase diagram was calculated via JmatProV9.0 software and referencing the corresponding literature. As shown in Figure 2, martensite, austenite, *M*_23_C_6_, Cu-enriched phase, G-phase and α-Cr phase were present in the MSS at 100–300 °C. Furthermore, the volume fraction of the Cu-enriched precipitate phases reached a maximum and stabilized at 600 °C. Moreover, the kinetics of Cu-enriched precipitation in martensite during tempering in a medium carbon steel were quantitatively measured via Jung’s work [19], and it was found that some solute Cu atoms had already precipitated after tempering over 450 °C. The amount of Cu-enriched precipitate was greatly enhanced at an elevated temperature of 600 °C [19]. Also, Yang et al. [20,21] investigated the effects of different temperatures (200–800 °C) on Cu precipitation kinetics in martensite of a low-carbon steel, revealing that the fastest precipitation of coherent BCC-Cu was obtained at ~600 °C. However, the rapid growth of Cu particles and the formation of FCC-Cu were detected at 670 °C [21]. According to Sun’s work [16], the coherent BCC-Cu precipitated during aging can enhance both strength and ductility simultaneously, while FCC-Cu exhibits a higher strengthening effect at the expense of ductility. Based on the above analysis, the as-deposited MSS specimens were tempered at 600 °C for 0.5 h, 1.0 h and 2.0 h, respectively.

### 2.3. Mechanical Properties Tests

The tensile samples were machined from the laser scanning direction (Figure 1b), with the dimensions illustrated in Figure 1c. The tensile test was conducted at a constant displacement rate of 0.2 mm/min using a PWS-E100 universal testing machine (Jinan Times Test Instrument Co., Ltd., Jinan, China). In order to ensure the repeatability and reliability of the results, the values of tensile properties were taken as the average of three tests. The MHV-2T microhardness tester (Changzhou Sanfeng Instrument Technology Co., Ltd., Changzhou China). was utilized for the microhardness measurement, applying a load of 200 g and a dwell time of 10 s. The microhardness value was determined as the average of five measurements.

### 2.4. Microstructural Characterization

For the microstructural analysis, the MSS specimens were mechanically ground and polished. In order to clearly reveal the microstructural features, the LDEDed specimens was etched in aqua regia for about 3 s. To characterize the phase compositions of the MSS specimens, a Miniflex600 X-ray diffractometer (XRD, SmartLab9kW; Rigaku Co., Ltd., Tokyo, Japan) was utilized with a step size of 0.02° and a source of Cu-Kα radiation at 40 kV and 40 mA. Microstructural observation was conducted using a MERLIN scanning electron microscope (SEM, Carl Zeiss AG, Oberkochen, Germany) fitted with an energy dispersive spectrometer (EDS). The size of each phase was measured using ImageJ2 software. A JEOL-2100 transmission electron microscope (TEM, FEI, Hillsboro, OR, USA) was used to examine the detailed microstructural features.

## 3. Results

### 3.1. Phase Constituent

**Figure 3:**
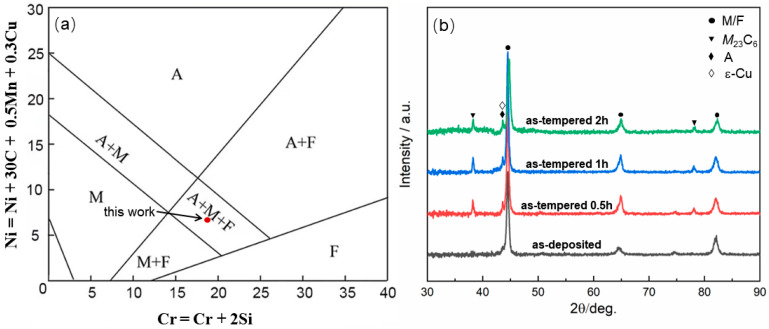
Schaeffler diagram and XRD spectra of the MSS specimens with different tempering time: (**a**) Schaeffler diagram; (**b**) XRD patterns.

Figure 3 illustrates the Schaeffler diagram and XRD pattern of LDEDed MSS specimens. The Cr_eq_ and Ni_eq_ values, representing chromium equivalent and nickel equivalent, are calculated using Schaeffler equivalent Formulas (1) and (2) as below, respectively [22]:

Cr_eq_ = Cr + 2Si, (1)

Ni_eq_ = Ni + 30C + 0.5Mn + 0.3Cu, (2)

where the respective weight percentage content (wt%) is used for each chemical element. The calculation results show that the Cr_eq_/Ni_eq_ value was 18.5/7.25, and thus the MSS specimen was composed of martensite (M), ferrite (F) and austenite (A). Figure 3b exhibits the XRD patterns of the MSS specimens with different tempering time. Obviously, the as-deposited MSS specimen primarily consisted of M/F, exhibiting similar diffraction peaks in the XRD pattern owing to their close lattice parameters. In contrast, the additional peaks of A, *M*_23_C_6_ and Cu-enriched (ε-Cu) phase occurred after the tempering treatment. This was consistent with the results of the equilibrium phase diagrams calculated via Jmatpro software (Figure 2). Furthermore, the intensity of M peaks decreased evidently by extending the tempering time, indicative of a promoted transition from martensite to austenite. Furthermore, the intensity of A/ε-Cu peaks increased in sequence with the extension of tempering time. Notably, the A and ԑ-Cu peaks were nearly overlapped because of their similar crystallographic parameters [23].

To clearly describe the variation of the A/ε-Cu peak and M/F peak in different states, the corresponding XRD peaks were fitted via a Gaussian function and the peak areas were estimated. The A/ε-Cu peak areas at ~43.6° of the as-deposited and as-tempered specimens for different tempering time (0.5 h, 1.0 h, 2.0 h) were 15.07, 41.77, 51.75 and 56.71, respectively. Comparatively, The M/F peak areas at ~44.5° were 423.79, 401.1, 398.4 and 386.59, respectively. This indicated that the A/ε-Cu peak intensity increased while the M/F peak intensity decreased for the MSS specimens with the increasing tempering time.

**Figure 4:**
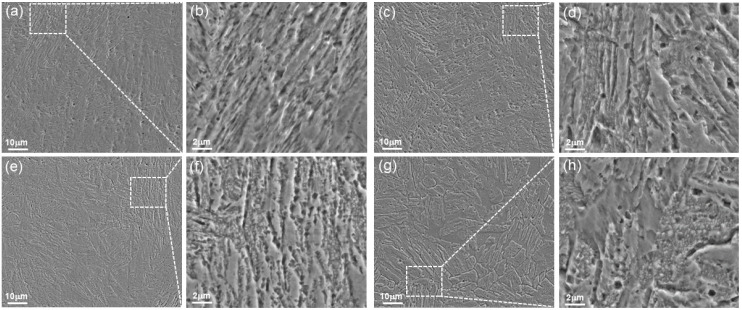
SEM morphology of the MSS specimens: (**a**,**b**) as-deposited, (**c**,**d**) as-tempered 0.5 h, (**e**,**f**) as-tempered 1.0 h and (**g**,**h**) as-tempered 2.0 h.

Figure 4 depicts the SEM images of the MSS specimens in different states. Apparently, the MSS specimens primarily consisted of lath martensite. Moreover, several precipitates can be observed inside the grain and at the grain boundary of martensite. Moreover, the number density of precipitated particles increased with the extension of tempering time, while the precipitated particles were significantly coarsened when the tempering time was 2.0 h (Figure 4h). This is due to the fact that the precipitated second-phase particle continued to aggregate and grow, generating a significant ripening process [24].

**Figure 5:**
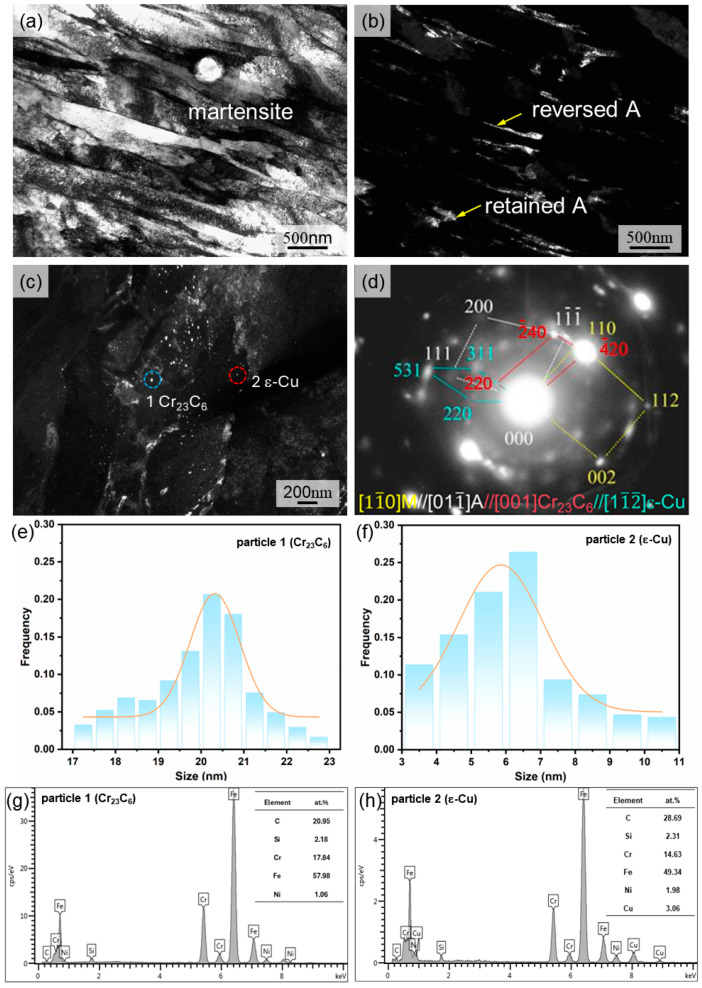
TEM images of the as-tempered 1.0 h MSS: (**a**) bright-field (BF) image of the lath M; (**b**) dark-field (DF) image of the retained A and reversed A; (**c**) DF image of nano-particles; (**d**) SAED pattern; (**e**) statistical results of the size of Cr_23_C_6_ particles; (**f**) statistical results of the size of ԑ-Cu particles; (**g**) EDS of particle 1 in (**c**); (**h**) EDS of particle 2 in (**c**).

Further detailed microstructure characterizations of the as-tempered 1.0 h MSS specimen were conducted via TEM (Figure 5). As seen in Figure 5a–c, the as-tempered 1.0 h MSS specimen was composed of lath martensite, austenite and nano-precipitates. Among them, the film-like and bulk austenite (A) was present in the lath martensite matrix. The incomplete martensitic transformation could be promoted because of the rapid cooling rates during LDED process together with the existence of the austenite-forming element Ni, leading to the appearance of bulk retained A [17,25]. The formation of reversed A during tempering process could be attributed to two aspects: (i) The synergistic effect between Cu and Ni. The diffusion of Ni can be promoted by the segregation of Cu at the martensite lath-boundaries by decreasing stacking fault energy, which can provide the nucleation energy for reversed A [26,27]. Also, the existence of Ni promotes the enrichment of Cu and thus facilitates the formation of reversed A [17,26]. (ii) The existence of retained A. The driving energy for reversed A nucleation can be alleviated by the retained A [28]. Consequently, the retained A and reversed A were observed in the as-tempered 1.0 h MSS. Furthermore, nano-sized precipitates were dispersed in the M matrix (Figure 5c). These particles were confirmed as the coexistence of ԑ-Cu and Cr_23_C_6_ via the SAED pattern (Figure 5d). Moreover, the particle 1 with the size of 17 to 23 nm (Figure 5e) and particle 2 with the size of 3 to 11 nm (Figure 5f) marked in Figure 5c was recognized as Cr_23_C_6_ carbide (Figure 5g) and ԑ-Cu (Figure 5h) via the EDS analysis, respectively.

In addition, considerable Cu-enriched nano-precipitates, having the size of 3 to 11 nm, can be observed in Figure 5c. During tempering at 600 °C for 1.0 h, the crystal structure of Cu-enriched nano-precipitates experienced the transformation of BCC-Cu→9R-Cu→FCC-Cu [16,27,29]. Herein, the coherent BCC-Cu was formed due to the Cu atoms accumulating in the martensite matrix, which improved the mechanical properties via coherent strengthening [16]. Moreover, the further enrichment of Cu atoms generated strain energy, leading to the movement of displacive atoms of BCC-Cu, and thus the 9R-Cu particles were formed [16]. Generally, 9R-Cu particles can be categorized into coherent and incoherent, which can enhance the strength through coherent strengthening and Orowan bypassing mechanisms, respectively [28]. It was well verified that 5 and 16 nm are the critical size for the crystallographic structure transitions from BCC-Cu to 9R-Cu and from 9R-Cu to FCC-Cu, respectively [16,19]. Therefore, the Cu-enriched nano-precipitates, exhibiting diameters of 3 to 11 nm in this work, can speculated to be the coexistence of coherent BCC-Cu and 9R-Cu. This finding is similar to Sun’s results [16], in which the BCC-Cu and 9R-Cu particles ranging from 4 to 12 nm were observed in the tempered low-carbon steel with a Cu content of 3.67 wt%.

Herein, the precipitation sequence of ԑ-Cu and Cr_23_C_6_ nano-particles can be explained by the diffusion coefficient of Cr (D_Cr-α_) and Cu (D_Cu-α_) in martensite. The formulas are as follows [12,30,31]:

D_Cr-α_ = 8.52exp(−59,900/RT), (3)

D_Cu-α_ = 300exp(−284,000/RT), (4)

where R and T represent the Boltzmann constant and diffusion temperature (K), respectively. The calculation results demonstrate that the D_Cr-α_ value (2.22 × 10^−3^ cm^2^/s) was much higher than D_Cu-α_ (3.07 × 10^−15^ cm^2^/s) at 600 °C, indicating that the Cr_23_C_6_ carbide was preferentially precipitated after tempering at 600 °C. Therefore, the Cr_23_C_6_ particles (17–22 nm) exhibited a larger size than that of ԑ-Cu nano-precipitate (3–11 nm). This was consistent with Jung’s work [19], revealing the Cr_23_C_6_ precipitation preceded the ԑ-Cu precipitation in Cu-bearing medium carbon steel during tempering at 450 to 600 °C.

### 3.2. Mechanical Properties

**Figure 6:**
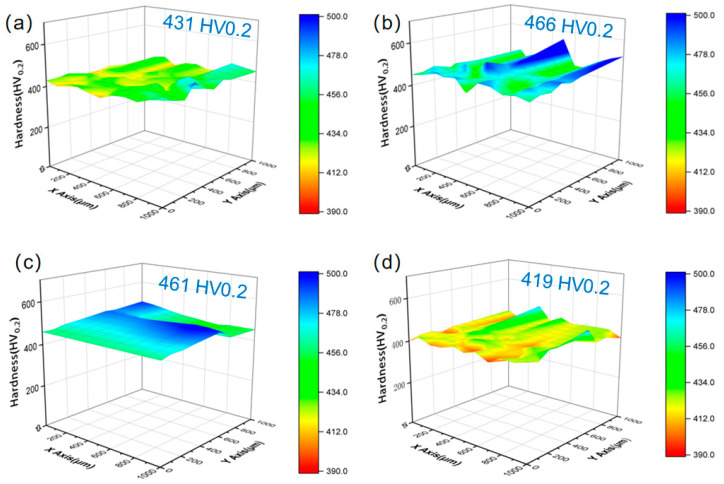
Microhardness cloud maps of the MSS specimens at different tempering times: (**a**) as-deposited; (**b**) as-tempered 0.5 h; (**c**) as-tempered 1.0 h; and (**d**) as-tempered 2.0 h.

Figure 6 presents the microhardness distribution cloud maps of LDEDed MSS specimens in different states. Notably, the microhardness of all the fabricated samples presents a negligible variation, suggesting a homogeneous microstructure. The average microhardness values of the MSS specimens for different tempering time (0.5 h, 1.0 h, 2.0 h) were 466 HV0.2, 461 HV0.2 and 419 HV0.2, respectively. Compared to the as-deposited MSS specimen (431 HV0.2), the microhardness of the as-tempered MSS specimens increased significantly with increasing tempering time of up to 1.0 h (Figure 6b,c), and then decreased with the time prolonging to 2.0 h (Figure 6d). This can be explained by the coarsened microstructure and increased austenite after 2.0 h tempering treatment. The MSS specimens presented the highest microhardness value of 466 HV0.2 after 0.5 h tempering treatment. Therefore, the microhardness of the LDEDed MSS specimen can be improved by tempering treatment.

**Figure 7:**
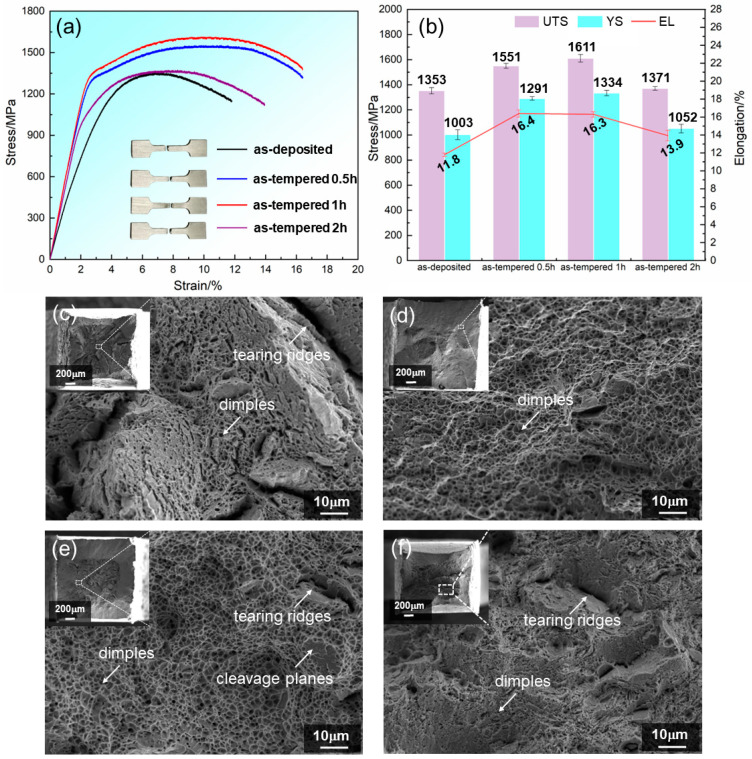
Tensile properties and fracture morphologies of the as-deposited and as-tempered MSS specimens. (**a**) Stress-strain curves; (**b**) comparison of tensile properties between as-deposited specimen and as-tempered specimens; (**c–f**) fractographs: (**c**) as-deposited, (**d**) as-tempered 0.5 h, (**e**) as-tempered 1.0 h and (**f**) as-tempered 2.0 h.

Figure 7a,b present the tensile curves and histogram of the MSS specimens with different tempering time. Clearly, the as-deposited MSS specimen exhibited a low tensile property with the UTS of 1353 MPa, YS of 1003 MPa and EL of 11.8%, respectively. Apparently, the strength and elongation significantly increased and reached the peak value after tempering for 1.0 h, with a remarkable enhancement to both strength (UTS: 1611 MPa, YS: 1334 MPa) and ductility (EL: 16.3%). Subsequently, the tensile properties decreased after an extending tempering treatment of 2.0 h. Noteworthily, the Young’s modulus E (i.e., the slope of the initial linear relation in the tensile curve) of the as-deposited MSS was determined as 35.93 GPa, which was relatively lower than that of the as-tempered MSS specimens (55.34 GPa, 56.38 GPa and 51.35 GPa for the different tempering times of 0.5 h, 1.0 h and 2.0 h, respectively, Figure 7a). This was due to the increased austenite and the reduced martensite after the tempering treatment (Figure 3), whose E values were 40–90 GPa and 20–50 GPa [32], respectively.

Figure 7c–f shows the fracture morphologies of the as-deposited and as-tempered MSS specimens. Apparently, all the LDEDed MSS specimens present significant necking deformation characterized by fiber zones and shear lips. In comparison to the as-deposited (Figure 7c) and as-tempered 2.0 h (Figure 7f) specimens, the necking degree and the shear lip size of the as-tempered 0.5 h (Figure 7d) and 1.0 h (Figure 7e) specimens were increased, demonstrating a higher ductility for the latter two counterparts. Comparatively, the fracture surfaces of the as-deposited specimen exhibited large tearing ridges and a small amount of dimples (Figure 7c), indicating a mixed brittle and ductile fracture mode. The formation of tearing ridges may be because the cracks intersect isolated microvoids ahead of cracks [33]. After a single-step tempering treatment, massive equiaxed dimples occurred in the fracture surfaces of the as-tempered 0.5 h (Figure 7d) and the as-tempered 1.0 h (Figure 7e) specimens, leading to a higher absorption energy during the plastic deformation process and thus a significant improvement in ductility [17]. However, the amount and size of the dimples decreased with the tempering time extending to 2.0 h (Figure 7f), indicating a reduction in ductility. These fractographs of the LDEDed specimens in different states are well consistent with the ductility variation as shown in Figure 7a,b.

Table 1 lists the UTS, YS and EL values of the as-deposited and as-tempered MSS obtained in the present work, and compared to those laser fabricated and hot forged 431 MSS from the literature [1,34]. Apparently, the Cu-bearing MSS displayed remarkably comprehensive mechanical properties after a simplified single-step tempering at 600 °C for 1.0 h, which is satisfactory for most industrial applications.

According to the above results, the superior combination of the strength and the ductility of the as-tempered 1.0 h MSS specimen can be ascribed to the following two aspects.

(i) Precipitates Cu-enriched phase. It is widely accepted that precipitation strengthening and coherent strengthening can be induced by the ԑ-Cu nano-precipitates [14,19]. After tempering at 600 °C × 1.0 h, the ԑ-Cu nano-precipitates are dispersed in the M matrix of the MSS specimen, which can effectively hinder the dislocation movement and thus enhance the strength of the MSS specimen via the dislocation multiplication and tangling [16,35]. Moreover, these ԑ-Cu precipitates (3–11 nm) exhibit a good coherent relationship with the M matrix, which can improve strength without sacrificing ductility. This can be attributed to the increase in surface energy and coherency strain during the shearing transformation of dislocations [34]. Furthermore, Sun et al. [16] reported that the ԑ-Cu precipitates can prevent premature crack initiation and thus improve plasticity in the tempered MSS with a Cu content of 3.67 wt%.
(ii) Enhanced reversed austenite (A). Noteworthily, the ductile reversed A can absorb the plastic deformation work, delay necking and thus enhance the ductility [17,36]. Furthermore, Jiang et al. [31] found that the elongation can be improved and the strength can be maintained after a 600 °C × 2.0 h tempering treatment for the hot-forged 3%Cu MSS, owing to the occurrence of reversed A induced by the synergistic effect of Cu and Ni. As discovered by Hu’s work [36], the high stability of reversed A facilitates the enhancement of tensile properties of the cast martensite steel after a 620 °C × 1.0 h tempering treatment. Thus, it could be concluded that the reversed A is favorable for improving both the strength and ductility of the tempered MSS specimens.

However, the microhardness and tensile properties of the specimen were slightly reduced when the tempering time was 2.0 h, which can be mainly associated with the coarsened microstructure and nano-precipitates. Generally, the coarsening of the precipitated phases can be caused by the Ostwald ripening process [24]. Due to the capillary effect, the smaller the precipitates are, the higher the solute concentration around them. In order to decrease the overall interface energy, the solute atoms can proceed the diffusion from the small precipitates to the large ones in response to the chemical potential resulted from the concentration gradient. Consequently, the number density of the small sized precipitates is decreased, whilst the retained precipitates are greatly coarsened [37].

## 4. Conclusions

In the present work, the Cu-bearing MSS with a high-strength and ductility was successfully prepared via the LDED technique. The influences of different tempering time at 600 °C on the microstructure and mechanical properties of the as-deposited 431-2.5Cu MSS specimen were studied. The following conclusions can be obtained:

(i) The as-deposited MSS mainly consisted of lath martensite, austenite and Cr_23_C_6_, while ԑ-Cu nano-precipitates appeared after a tempering treatment. With the extension of tempering time, the fractions of austenite and ԑ-Cu precipitates were increased correspondingly.
(ii) Owing to the synergistic effects of precipitation strengthening, coherent strengthening and softening caused by the generation of reversed austenite, the strength and elongation can be enhanced with an increase in tempering time up to 1.0 h, and then decreased when the tempering time is 2.0 h.
(iii) The as-deposited 431-2.5Cu specimen exhibits low mechanical properties with the microhardness of 431 HV0.2, UTS of 1353 MPa, YS of 1003 MPa and EL of 11.8%. After the optimized single-step tempering treatment (600 °C × 1.0 h), the LDEDed 431-2.5Cu specimen demonstrated superior mechanical properties, including a microhardness of 461 HV0.2, UTS of 1611 MPa, YS of 1334 MPa and EL of 16.3%, respectively.

This work will have great potential for facilitating wide industrial applications of Cu-bearing MSS with ultra-high strength and ductility.

## Figures and Tables

**Table 1:** Tensile properties of Cu-bearing MSS specimens in different states and AISI 431 MSS prepared via LDED and wrought method reported in the literature.

Method	Specimens	UTS /MPa	YS /MPa	EL /%	Post-Heat Treatment	Reference
LDED	431–2.5Cu	1353 ± 25	1003 ± 39	11.8 ± 0.2	As-deposited	This work
	431–2.5Cu	1551 ± 19	1291 ± 14	16.4 ± 0.4	Tempering (600 °C × 0.5 h)	
	431–2.5Cu	1611 ± 31	1334 ± 21	16.3 ± 0.3	Tempering (600 °C × 1.0 h)	
	431–2.5Cu	1371 ± 15	1052 ± 34	13.9 ± 0.6	Tempering (600 °C × 2.0 h)	
LDED	431 MSS	905 ± 6	-	16.3 ± 0.8	Tempering (680 °C × 2.0 h)	[1]
LDED	431 MSS	1283 ± 16	-	14.5 ± 1.5	Tempering (680 °C × 2.0 h) + Solid-solution (1050 °C × 45 min) + Tempering (315 °C × 3.0 h)	[1]
Hot forged	431 MSS	1645	1080	13	Annealing (640 °C × 2.0 h) + Solid solution (1070 °C × 0.5 h)+ Tempering (200 °C × 1.0 h)	[34]

## Data Availability

The original contributions presented in the study are included in the article, further inquiries can be directed to the corresponding authors.
